# Varicella-zoster virus infection in pregnancy: a case of aseptic meningitis before 20 weeks gestation and review of the literature

**DOI:** 10.3389/fmed.2025.1631412

**Published:** 2025-08-08

**Authors:** Gao Song, Meng-Qun Cheng, Nan Yang, Rong Li, Cai-Qiong Zhang, Si-Wei Chen

**Affiliations:** ^1^Department of Pharmacy, Puer People's Hospital, Pu’er, China; ^2^Department of Reproductive Medicine, Puer People's Hospital, Pu’er, China; ^3^Department of Neurology, Puer People's Hospital, Pu’er, China

**Keywords:** varicella-zoster virus, pregnancy, aseptic meningitis, antiviral therapy, multidisciplinary management, preterm delivery

## Abstract

**Background:**

Varicella zoster virus (VZV) infection during pregnancy can lead to severe complications. However, cases complicated by aseptic meningitis in early pregnancy (<20 weeks) are extremely rare, and clinical management and pregnancy outcomes remain unclear.

**Case report:**

We report a case of a 36-year-old pregnant woman who developed right upper abdominal pain, headache, and cough at 17 weeks of gestation, followed by clustered herpes zoster lesions in the right lumbar region and signs of meningeal irritation. Aseptic meningitis was confirmed by cerebrospinal fluid analysis (lymphocytes 184 × 10^6^/L, protein 938 mg/L), and other etiologies were excluded by imaging. A 14-day course of acyclovir (5 mg/kg q8h) combined with prednisone achieved complete symptom resolution through a multidisciplinary collaboration (neurology, obstetrics, infectious diseases, pharmacy, and nursing). The patient experienced unexplained preterm delivery at 35 weeks, delivering a healthy male infant (2,680 g, Apgar 9–10-10) via cesarean section, with no sequelae at the 1-year follow-up.

**Conclusion:**

This represents the fourth reported case of VZV meningitis during pregnancy globally and the first occurring before 20 weeks of gestation with preterm delivery as a unique outcome. Key implications: (1) Early-to-mid gestation VZV infection may affect pregnancy outcomes through distinct mechanisms; (2) multidisciplinary management is critical for optimizing maternal-fetal prognosis; and (3) further investigation is needed to elucidate the relationship between infection timing and preterm birth.

## Introduction

Varicella-zoster virus (VZV), a member of the human herpesvirus family (HHV-1 to HHV-8, including herpes simplex virus type 1/2, varicella-zoster virus, Epstein–Barr virus, and cytomegalovirus), is classified under the Alphaherpesvirinae subfamily. It is a double-stranded DNA virus with neurotropic and dermatropic properties that is primarily transmitted through respiratory droplets or direct contact with skin lesions. Global epidemiological data indicate that VZV causes approximately 140 million cases of varicella annually, of which 4.2 million develop severe complications, resulting in 4,200 deaths (1). Notably, VZV infection during pregnancy presents a unique clinical challenge. Due to pregnancy-specific immunomodulatory mechanisms (e.g., Th1/Th2 balance shift) (2–5), pregnant individuals face significantly increased risks of severe complications, such as pneumonia and encephalitis. More critically, the virus can be vertically transmitted via the placenta, leading to congenital varicella syndrome in approximately 2% of fetuses infected between 13–20 weeks of gestation, manifesting as limb hypoplasia, neurological damage, and other malformations (6).

Among the various complications of VZV infection during pregnancy, meningitis is rare. According to existing literature, only three cases of VZV meningitis during pregnancy have been reported globally (7–9), including two in immunocompetent pregnant individuals and one in a human immunodeficiency virus (HIV)-positive patient with immunodeficiency. This article presents a rare case of VZV-associated aseptic meningitis at 17 weeks of gestation using a systematic literature review to address the following key issues: (1) differences in clinical features and management approaches for VZV infection across gestational stages; (2) the critical role of multidisciplinary collaboration (encompassing neurology, obstetrics, infectious diseases, pharmacy, and nursing teams) in improving outcomes for pregnancy-associated neurological infections; and (3) the potential relationship between the gestational timing of infection and adverse pregnancy outcomes (e.g., preterm delivery), along with underlying pathophysiological mechanisms. This study aimed to provide evidence-based guidance for clinical practice and bridge the theoretical gaps in the management of this special population.

### Case presentation

The patient was a 36-year-old gravida 3 and para 1 (G3P1) woman (with a history of cesarean section in February 2019 due to placenta previa bleeding), who was previously healthy with no documented history of varicella/herpes zoster infection or vaccination. At 17 weeks of gestation, she was admitted with a chief complaint of “sudden right upper abdominal pain for 7 h, accompanied by headache and cough.” Abdominal pain was paroxysmal without radiation and was associated with a mild headache and nonproductive cough. After 3 days of empirical treatment (anti-infectives, antispasmodics, and analgesics) combined with cerebral dehydration therapy (Supplementary Table S1), the abdominal pain resolved, but low-grade fever persisted (temperature fluctuating between 36.8–37.6°C) with progressively worsening bilateral throbbing temporal headache, prompting transfer to neurology. Neurological examination revealed signs of meningeal irritation (nuchal rigidity and positive Kernig’s sign) with no history of migraine. Dermatological examination revealed clustered herpes zoster lesions in the right lumbar region.

#### Investigation

Obstetric ultrasound showed fetal development consistent with gestational age, whereas abdominal ultrasound revealed multiple gallstones. Laboratory tests demonstrated: white blood cells 9.5 × 10^9^/L (reference range: 3.5–9.5 × 10^9^/L) with 77.3% neutrophils, hemoglobin 98 g/L (reference range: 115–150 g/L), and platelets 208 × 10^9^/L (reference range: 125–350 × 10^9^/L). Serological tests for HIV, hepatitis B (HBsAg/HBeAg), and syphilis (chemiluminescence assay) were negative. Additional testing revealed negative results for *Mycoplasma pneumoniae* IgM, influenza A/B antigen, anti-herpes simplex virus I/II IgM, anti-cytomegalovirus IgM, and anti-rubella IgM.

Brain Magnetic resonance imaging (MRI), magnetic resonance angiography (MRA), and magnetic resonance venography (MRV) revealed no meningeal enhancement, hydrocephalus, vascular abnormalities, or hemorrhage but indicated maxillary sinusitis (Supplementary Figures S1, S2). Initial lumbar puncture showed: opening pressure 150 mmH₂O, cerebrospinal fluid (CSF) white blood cells 184 × 10^6^/L (reference range: 0–8.0 × 10^6^/L) with 97.3% lymphocytes, protein 938 mg/L (reference range: 150–450 mg/L), glucose 2.25 mmol/L (reference range: 2.22–3.89 mmol/L), chloride 119.2 mmol/L (reference range: 120–132 mmol/L), and markedly elevated total immunoglobulins IgA/IgM/IgG (Supplementary Table S2). CSF Gram, acid-fast, and India ink stains were negative. After multidisciplinary consultation (infectious diseases, dermatology, obstetrics, hepatobiliary surgery, and pharmacy), common pathogens including bacteria, fungi, and *Mycobacterium tuberculosis* were excluded. Although VZV DNA Polymerase chain reaction (PCR) was not performed due to patient refusal of self-paid testing, the final clinical diagnosis was herpes zoster (right lumbar segment) with VZV meningitis.

#### Treatments

Antibiotics were discontinued and intravenous acyclovir (5 mg/kg q8h) was initiated. After 4 days of treatment, repeat lumbar puncture showed a CSF pressure of 140 mmH₂O, cerebrospinal fluid white blood cells increased to 315 × 10^6^/L, and total immunoglobulins decreased from previous levels (although still above the normal range) (see Supplementary Table S1). Prednisone (10 mg qd) was added as anti-inflammatory therapy.

#### Outcome and follow-up

**Figure 1:**
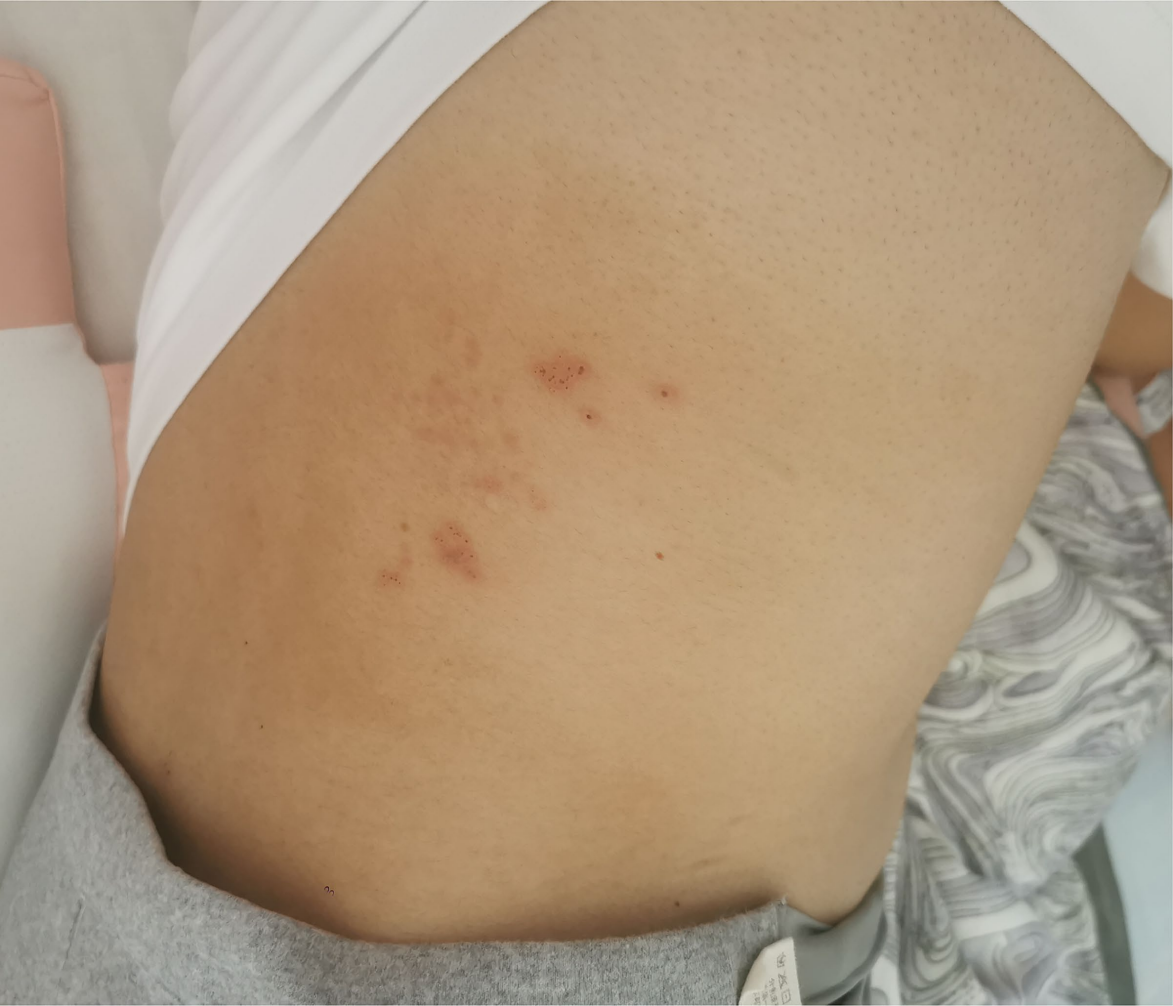
Healing status of herpes zoster skin lesions in the lumbar region before hospital discharge following antiviral treatment.

After completing the 14-day acyclovir course and 10-day corticosteroid therapy, the patient’s lumbar herpes zoster lesions gradually crusted (Figure 1), with complete resolution of headache and no neurological deficits. At discharge, cerebrospinal fluid analysis showed that the white blood cell count had decreased to 75 × 10^6^/L (CSF pressure 130 mmH₂O) and normalized protein levels. Post-discharge fetal chromosomal testing at Yunnan Provincial First People’s Hospital revealed no significant abnormalities in the G-banded karyotype analysis of amniotic fluid cells at the 400-band level, with normal fetal development during subsequent prenatal follow-up.

At 35^+1^ weeks gestation, the patient underwent cesarean delivery (through the previous uterine incision) for “threatened preterm labor,” delivering a male infant (2,680 g, Apgar scores 9–10-10) with grossly normal placenta. One-year neonatal follow-up revealed no signs of congenital varicella syndrome.

## Discussion

Humans are currently the only known natural hosts of VZV infections. VZV manifests clinically in two distinct forms, varicella (chickenpox) and herpes zoster (shingles). The virus can travel retrogradely along sensory nerve axons or be transported via infected T cells that fuse with neuronal cells, ultimately establishing latency in the dorsal root ganglia or cranial nerve ganglia. When host immunity declines, VZV-specific cellular immunity decreases, allowing latent viruses to reactivate, replicate extensively, and migrate along sensory nerve axons to the skin, causing herpes zoster in the corresponding dermatome.

Varicella is highly contagious, with primary infections typically causing symptomatic chickenpox in susceptible individuals. Most pediatric cases are relatively mild and self-limiting. However, varicella can also lead to severe complications including soft tissue infections, pneumonia, hepatitis, Reye’s syndrome, and meningitis. High-risk populations (e.g., adolescents, adults, pregnant women, and immunocompromised individuals) face a significantly increased risk of severe complications.

Although VZV infection during pregnancy is relatively uncommon, it carries substantial risk. Estimates suggest approximately 4–7 per 10,000 pregnant women contracted varicella in the pre-vaccine era (10). Since the introduction of the varicella vaccine in the United States in 1995, its epidemiology has changed. A study of 7.7 million hospitalized pregnant women demonstrated a decreased incidence, documenting 935 VZV-related hospitalizations (1.21 cases per 10,000 pregnancies; 95% CI 1.13–1.29) (11). While these data reflect the vaccine’s positive impact, pregnant women with varicella still face higher complication and mortality risks than non-pregnant adults. Research indicates (12) that 10–20% of pregnant women with varicella may develop pneumonia, with unpredictable clinical progression potentially rapidly deteriorating to hypoxia and respiratory failure, significantly increasing the maternal mortality risk. Although vaccination has markedly reduced clinical disease, complications and breakthrough infections still occur. Recent data from a large U. S. inpatient database (11) revealed that among 935 pregnant women diagnosed with VZV infection, 2.5% developed VZV pneumonia with no reported mortality.

Pregnancy involves complex immunomodulatory processes that maintain tolerance of the semi-allogeneic fetus (13, 14). This is generally characterized by a state of moderate systemic immunosuppression, particularly affecting cell-mediated immunity (with relative suppression of Th1-type responses and enhancement of Th2-type responses) (15, 16). Such adaptation may increase maternal susceptibility to primary infection by certain viruses (including VZV) or the reactivation of latent viruses (e.g., VZV). During the second trimester, when the present case occurred, this immunological adaptation became relatively stable, yet may compromise the control of newly acquired or reactivated VZV infections, potentially elevating the risk of disseminated or neurological complications. However, the precise relationship between these immunological changes and the risk of specific VZV complications requires further investigation.

This case highlights the unique clinical challenges of VZV-associated aseptic meningitis during early-to mid-pregnancy. The patient had no history of vaccination or prior infection. Although the clinical presentation was consistent with typical herpes zoster, the absence of pre-infection serological data makes it impossible to completely rule out atypical primary infections. In this case, the classic unilateral dermatomal distribution of painful clustered vesicles is highly characteristic of herpes zoster (VZV reactivation), distinguishing it from other viral infections (e.g., herpes simplex, which typically recurs at mucocutaneous junctions without dermatomal patterning), bacterial skin infections (e.g., impetigo with prominent purulent discharge), and allergic rashes in terms of morphology and distribution.

Notably, this patient experienced preterm delivery at 35 weeks, contrasting sharply with the term deliveries reported in the literature, suggesting that early pregnancy VZV infection may affect pregnancy outcomes through immune-mediated mechanisms or subclinical placental inflammation. Although VZV-related encephalitis and aseptic meningitis are among the most severe neurological complications (occurring less frequently than VZV pneumonia (17)), their clinical outcomes differ significantly: encephalitis has a mortality rate of up to 10%, with 15% of survivors experiencing neurological sequelae (18), whereas patients with VZV meningitis typically recover completely without neurological deficits after standard antiviral therapy (~2 weeks). This case and other reports demonstrate that such patients exhibit only meningeal involvement without parenchymal invasion and achieve favorable post-treatment outcomes (Table 1). It is important to note that VZV infection can also cause other rare neurological complications (e.g., focal neurological deficits and transverse myelitis) (19), and central nervous system (CNS) symptoms may precede characteristic skin eruptions (20), further complicating clinical diagnosis.

**Table 1:** Summary of reported cases of varicella-induced aseptic meningitis during pregnancy, with clinical features, treatments, and outcomes.

Year	Author	Age (yrs)	Gestation (wks)	Febrile	VZV history	Symptoms	Immunity status	complicating disease	Initial CSF analysis	Follow-up CSF analysis	Imaging findings	Treatment, recovery	Outcome
2024	Gao (This case)	36	17	Y	NK	low back pain, headache, neck stiffness	normal	Herpes zoster on the lower back	WBC 184×10^6/L (97.3% lymphocytes), Protein 938 mg/L, Chloride 119.2 mmol/L	^*^Day 4: WBC 315×10^6/L, Protein high; Day 13: WBC 75×10^6/L, normalized	Normal MRA/MRV; sinusitis	IV acyclovir 5 mg/kg q8h 14d, Oral prednisone 10 mg qd 10d; Recovery after 14d	Preterm delivery (35 wks), mother and child healthy
2020	Mroue	27	25	N	NK	Severe headache, nausea, neck stiffness, no rash	low	Diabetes	WBC 12×10^6/L, Protein 220 mg/L, Glucose 88 mg/dL	NK	Normal MRI/MRA/MRV	IV acyclovir 5-10 mg/kg q8h 14d; Recovery after 14 days	Term delivery, healthy neonate
2020	Gholkar	27	32	N	Y	Throbbing headache, vomiting, no rash	normal	Epilepsy controller	WBC 365×10^6/L (100% lymphocytes), Protein 1 g/L	VZV PCR negative	Normal MRA/MRV	IV acyclovir for 2 weeks	Term delivery, healthy neonate
2008	Jayakrishnan	36	28	Y	NK	Hip pain, headache, stiff neck, blistering rash	low	HIV	VZV PCR positive, no bacterial microorganisms	NK	Normal CT	IV acyclovir 10 mg/kg q8h 7d, oral valaciclovir continuation	Term delivery, HIV-negative neonate

VZV, varicella zoster virus; HIV, human immunodeficiency virus; CSF, cerebrospinal fluid; PCR, polymerase chain reaction; IV, intravenous; NK, not known; WBC, white blood cell count; CT, computed tomography; MRI, magnetic resonance imaging; MRA, magnetic resonance angiography; MRV, magnetic resonance venography; Y, yes; N, no.

*After treatment with acyclovir.

Congenital Varicella Syndrome (CVS) was first described by Lunk> t and Lynch in 1947. A prospective study (*n* = 1,739) demonstrated that maternal varicella infection before 12 weeks of gestation carries a 0.4% risk of fetal congenital malformations, increasing to approximately 2% for infections occurring at 13–20 weeks (6). CVS has a mortality rate of up to 30% within the first months of life, with 15% of survivors developing herpes zoster by the age of 4 (6). Current evidence suggests that this increased risk is primarily mediated by transplacental viral infection of fetal tissues during organogenesis, where viral interference with cellular proliferation/differentiation may cause multisystem abnormalities (e.g., limb hypoplasia, cutaneous scarring, and ocular/neurological damage). Secondary mechanisms such as maternal inflammatory responses and placental dysfunction may also contribute. Although the overall risk of intrauterine VZV transmission is low (<2%) and typically benign (21), theoretical concerns remain that viral reactivation from latent sensory ganglia during pregnancy could enable placental transmission (22).

Regarding VZV infection and pregnancy outcomes, existing studies indicate that varicella generally does not increase spontaneous abortion risk (2). Multiple investigations (23, 24) have found no statistically significant differences in spontaneous abortion, preterm delivery, or stillbirth rates between varicella-infected pregnancies and controls, although these studies were limited by small sample sizes. Notably, Pastuszak et al. (25) reported that varicella before 20 weeks may elevate the risk of elective termination and preterm birth. In our case, preterm delivery at 35 weeks following VZV meningitis at 17 weeks contrasts sharply with literature-reported term deliveries in post-20-week infections (Table 1), suggesting that early pregnancy VZV may disrupt pregnancy maintenance through distinct mechanisms. Potential pathways include (1) maternal inflammatory responses (VZV-induced cytokines affecting uterine contractility) and (2) placental dysfunction (localized viral inflammation). These hypotheses require validation through (1) systematic collection of pregnancy outcomes by infection timing, (2) placental histopathological/molecular analyses, and (3) longitudinal monitoring of maternal-fetal inflammatory markers, which are essential for elucidating VZV-associated preterm delivery mechanisms.

The safety and efficacy of antiviral therapy during pregnancy remain critical considerations in clinical decision making. Observational studies have demonstrated that acyclovir use in early pregnancy is safe, with no significant increase in congenital malformations (26, 27). However, specific dosing recommendations for antiviral administration before 20 weeks gestation remain insufficient. Our approach, based on prior case reports and multidisciplinary consultation, adopted cautious dose adjustments to balance maternal recovery with fetal safety. Randomized controlled trials are urgently needed to generate high-quality evidence to support the development of clinical guidelines.

### Diagnostic basis

The diagnosis of VZV-associated aseptic meningitis in this case was established through a comprehensive evaluation of clinical manifestations, laboratory findings, and imaging characteristics. The patient initially presented with classic dermatomal herpes zoster (clustered vesicles in the right lumbar region), followed by temporally correlated meningeal symptoms (headache and neck stiffness) and CSF alterations typical of viral meningitis (lymphocytic pleocytosis, markedly elevated protein with normal glucose levels). Systematic CSF analysis, serological testing, and neuroimaging excluded bacterial, tuberculous, fungal, and other viral (e.g., HSV, CMV, and rubella) meningitis. Rapid clinical improvement and CSF parameter normalization following acyclovir therapy further supported the VZV infection.

Although CSF VZV DNA PCR remains the diagnostic gold standard, in resource-limited settings or when patients decline testing, clinical diagnosis based on characteristic rash and neurological symptoms, as endorsed by the ICD-11 criteria and Chinese Expert Consensus on Herpes Zoster Diagnosis and Treatment (28), holds significant value. This case demonstrates that reliable clinical diagnosis remains feasible for patients with typical dermatomal rashes and neurological symptoms, even without direct pathogen confirmation. However, future studies should prioritize pathogen detection to enhance diagnostic accuracy.

### Study limitations

This study had several limitations that warrant acknowledgment. First, we were unable to confirm the etiology through VZV PCR testing because the patient refused to undergo external laboratory testing. Second, pretreatment photographic documentation of skin lesions was unavailable. Additionally, VZV-specific antibody testing for newborns has not yet been completed. These limitations primarily result from the constraints in real-world clinical practice.

### Key implications

This study highlights several critical clinical insights: (1) while exceptionally rare, VZV-associated aseptic meningitis can occur during early to mid pregnancy (<20 weeks), necessitating clinician awareness; (2) multidisciplinary team management (integrating neurology, obstetrics, infectious diseases, and nursing expertise) proved vital for optimal outcomes; (3) preterm delivery at 35 weeks suggests that early/mid-gestation VZV infection may uniquely impact pregnancy outcomes (vs. late-gestation cases), warranting investigation of placental pathology and immune mechanisms. (4) The low-dose acyclovir/steroid regimen demonstrated therapeutic efficacy without adverse effects. (5) These findings reinforce the preventive importance of pre-conception VZV seroscreening and vaccination.

## Data Availability

The original contributions presented in the study are included in the article/Supplementary material, further inquiries can be directed to the corresponding author/s.
