# Supplementary material for: Varicella-zoster virus infection in pregnancy: a case of aseptic meningitis before 20 weeks gestation and review of the literature
Source: Front Med (Lausanne). 2025 Aug 8;12:1631412. doi: 10.3389/fmed.2025.1631412 (PMC12370731; doi:10.3389/fmed.2025.1631412)

Table S1 Summary of Major Drug Treatments

| **Start Date** | **End Date** | **Drug Name** | **Dosage and Administration** |
| --- | --- | --- | --- |
| 4.7 | 4.10 | Cefoperazone-Sulbactam Injection | 1.5g q8h iv.gtt |
| 4.8 | 4.10 | Dydrogesterone Tablets | 20mg bid po |
| 4.6 | 4.9 | Phloroglucinol Injection | 80mg bid im |
| 4.6 | 4.6 | Raceanisodamine Injection | 10mg st iv.gtt |
| 4.9 | 4.9 | Mannitol Injection | 125ml bid iv.gtt |
| 4.11 | 4.25 | Acyclovir Injection | 5mg/kg q8h iv.gtt |
| 4.16 | 4.25 | Prednisone Tablets | 10mg qd po |
| 4.25 | 4.26 | Prednisone Tablets | 5mg qd po |

Note：iv.gtt：intravenous guttae；po：per os；im：intramuscular；st：statim；q8h：quaque 8 hora；bid：bis in die；qd：quaque die

Table S2 Cerebrospinal Fluid Total Immunoglobulin(Ig) Test Results

| Date | Total IgA(Reference range: 0-2.0 mg/L) | Total IgG(Reference range: 4.8-58.6 mg/L) | Total IgM(Reference range: 0.19-0.29 mg/L) |
| --- | --- | --- | --- |
| 4.10 | 12.5 | 85.9 | 4.42 |
| 4.14 | 9.2 | 66.5 | 3.99 |

Note：Ig: Immunoglobulin

Figure S1 Results of Conventional Cranial MRI Sequences

T1 - Weighted Imaging (T1): Displays the T1 - weighted signal characteristics of cranial tissues at different levels, reflecting the differences in longitudinal relaxation of tissues.

T2 - Weighted Imaging (T2): Shows the T2 - weighted signal manifestations of the cranium at multiple levels, reflecting the differences in transverse relaxation of tissues.

Fluid - Attenuated Inversion Recovery Sequence (FLAIR): Suppresses the signal of free water and presents the FLAIR signal characteristics of cranial parenchyma and lesions at multiple levels.


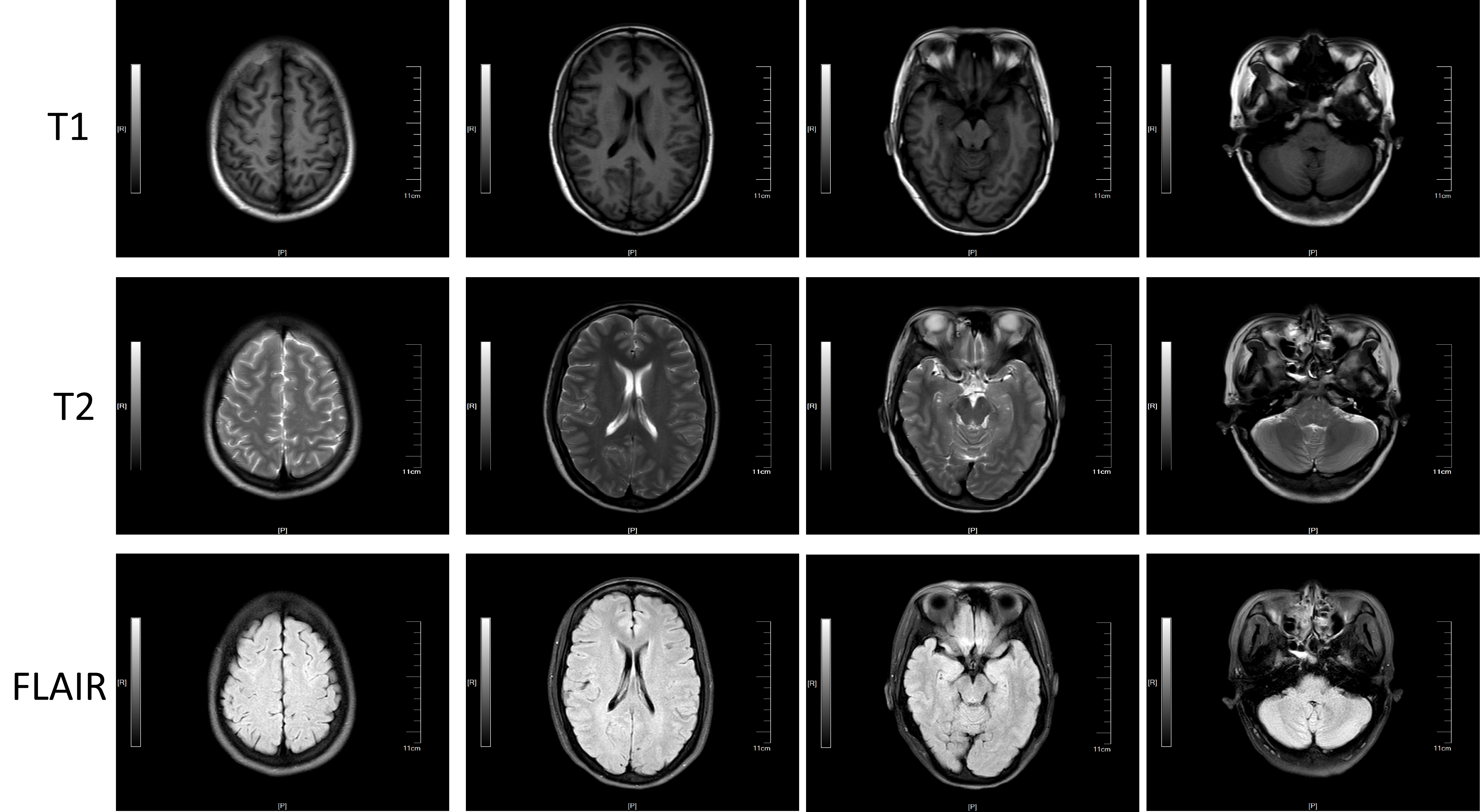


Figure S2 Results of Cranial Vascular Imaging

(A - B) Magnetic Resonance Venography (MRV): A is the coronal plane and B is the sagittal plane, showing the morphology, course, and distribution of the cranial venous system.

(C - D) Magnetic Resonance Angiography (MRA): C and D are axial planes, presenting the structure, branches, and blood perfusion of the cranial arterial system.


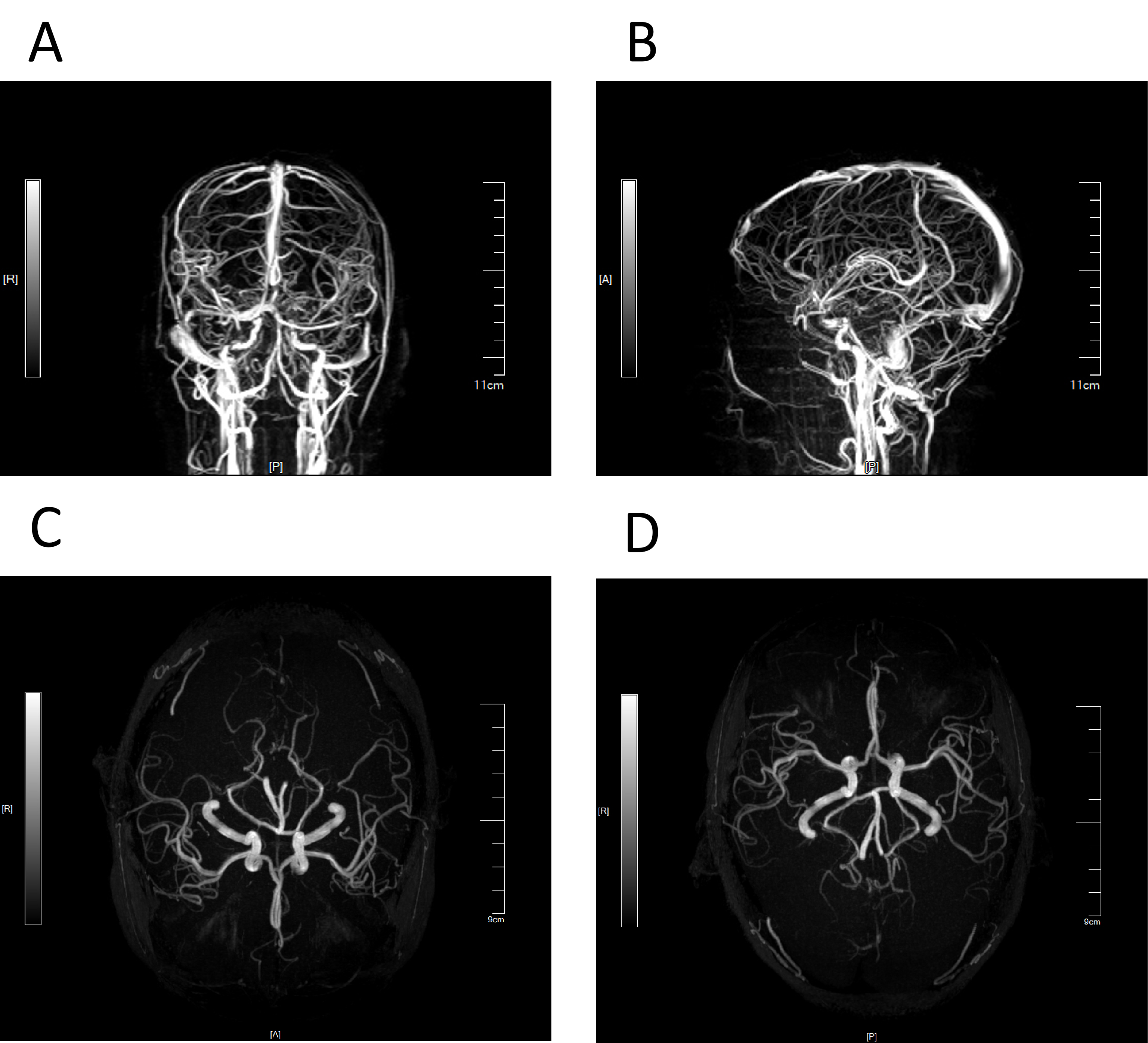

Supplement: Supplementary file 1 [file Table_1.DOC]
